# Endogenous and Exogenous Antioxidants in Skeletal Muscle Fatigue Development during Exercise

**DOI:** 10.3390/antiox12020501

**Published:** 2023-02-16

**Authors:** Elżbieta Supruniuk, Jan Górski, Adrian Chabowski

**Affiliations:** 1 Department of Physiology, Medical University of Białystok, 15-222 Białystok, Poland; 2 Department of Medical Sciences, Academy of Applied Sciences, 18-400 Łomża, Poland

**Keywords:** muscle fatigue, exercise, oxidative stress, antioxidants

## Abstract

Muscle fatigue is defined as a decrease in maximal force or power generated in response to contractile activity, and it is a risk factor for the development of musculoskeletal injuries. One of the many stressors imposed on skeletal muscle through exercise is the increased production of reactive oxygen species (ROS) and reactive nitrogen species (RNS), which intensifies as a function of exercise intensity and duration. Exposure to ROS/RNS can affect Na^+^/K^+^-ATPase activity, intramyofibrillar calcium turnover and sensitivity, and actin–myosin kinetics to reduce muscle force production. On the other hand, low ROS/RNS concentrations can likely upregulate an array of cellular adaptative responses related to mitochondrial biogenesis, glucose transport and muscle hypertrophy. Consequently, growing evidence suggests that exogenous antioxidant supplementation might hamper exercise-engendering upregulation in the signaling pathways of mitogen-activated protein kinases (MAPKs), peroxisome-proliferator activated co-activator 1α (PGC-1α), or mammalian target of rapamycin (mTOR). Ultimately, both high (exercise-induced) and low (antioxidant intervention) ROS concentrations can trigger beneficial responses as long as they do not override the threshold range for redox balance. The mechanisms underlying the two faces of ROS/RNS in exercise, as well as the role of antioxidants in muscle fatigue, are presented in detail in this review.

## 1. Introduction

Substantial evidence has suggested that regular physical activity of a moderate intensity, which lasts for at least 30 min each day, is beneficial for the maintenance of good health and reductions in potential disease risks [1]. However, unaccustomed or exhaustive exercise can result in detrimental health effects such as muscle damage, inflammation and increased oxidative stress. In this respect, muscle fatigue is a commonly experienced phenomenon referred to as a failure at any point upstream of cross-bridges that not only limits athletic performance but also restricts daily activity under numerous pathological conditions, including neurological and muscular diseases, as well as aging and frailty. Exercise-induced muscle fatigue can occur under a submaximal and maximal exercise intensity, and it engages either single limbs or the whole body [2].

Skeletal muscle’s ability to regenerate and remodel in response to metabolic changes brought on by exercise is crucial to contractile function. One of the challenges faced by contracting muscle is a rise in reactive oxygen/nitrogen species (ROS/RNS) content, which is a necessary prerequisite for the control of the signaling pathways associated with fatigue delay. Experimental observations of ROS/RNS-driven deterioration in muscle performance [3,4] encourage the use of exogenous antioxidants to counteract oxidative stress and enhance force generation. Years of extensive studies have provided data to support both the beneficial health-effects of antioxidant treatment and the potentially deleterious consequences of antioxidant supplementation that override potential benefits. Indeed, pretreatment with ROS scavengers was found to reduce the extent of fatigue in isolated muscle fibers [5] and in humans. For example, NAC supplementation in well-trained athletes, preceding strenuous physical training, improved redox balance and promoted adaptive processes [6]. Other evidence indicates that antioxidants enhance muscle recovery following intense muscle-damaging exercise [7]. However, the use of antioxidants may lead to diminutions in prominent physiological cellular signals that depend on the presence of ROS/RNS and are essential in the development of skeletal muscle adaptations to exercise [8]. Therefore, the awareness of the mechanisms underlying the transition from beneficial effects to an increase in muscle fatigue and delayed recovery stands as a critical point in ROS/RNS research [9,10]. Moreover, some data indicate that excessive antioxidant administration might paradoxically enhance oxidative stress, an idea supported by a study investigating the intake of vitamin C in the course of three-week training program. Antioxidant ingestion elevated certain oxidative markers, such as catalase and protein carbonyls at rest and superoxide dismutase (SOD) following exercise bouts [11], suggesting that vitamins act as prooxidants in excessive concentrations. Therefore, a considerable debate regarding the beneficial consequences of exogenous antioxidants has reinforced adjustments in strategies undertaken to counteract fatigue. In this review, we focus on recent advances in molecular mechanisms engaged in ROS/RNS signaling and antioxidant effects in redox-sensitive adaptations. We intend to verify whether there is sufficient evidence to support antioxidant supplementation in order to attenuate muscle fatigue, as well as to indicate critical factors in the optimization of training protocols and beneficial ROS/RNS-triggered outcomes.

## 2. Peripheral Muscle Fatigue

The progressive reduction in muscle fibers’ ability to generate force originates at different levels of the motor system and can be categorized into two types, namely, central and peripheral fatigue [12]. Peripheral mechanisms of fatigue refer to activity-induced mechanical failure through processes at or distal to neuromuscular junctions, so they can be attributed to neuromuscular transmission and excitation–contraction coupling. Additionally, muscle bioenergetics, which can be understood as changes in energy demand, the accumulation of metabolites, or the depletion of fuels, provides control signals for the regulation of muscle fatigue. From a biochemical point of view, metabolic acidosis (a drop of pH up to 6.2–6.5 during maximal contractions) is related to the function of each ATP-producing system, including phosphagen, glycolysis, and mitochondrial respiration, and the consequent accumulation of inorganic phosphates (Pi), lactate and H^+^. In humans, metabolic acidosis was shown to impair muscle’s ability to sustain submaximal force, but it did not exert an inhibitory effect on maximal isometric-force output [13]. H^+^ contributes to fatigue by affecting ATP generation, calcium (Ca^2+^) release from the sarcoplasmic reticulum, and depressing actin affinity to myosin, thus reducing the number of cross-bridges and inhibiting muscle’s ability to produce force and motion [14]. Physical exercise is also a physiological factor that impacts the oxidant–antioxidant balance through the enhanced generation of ROS/RNS. Under normal circumstances, cells maintain redox homeostasis by matching the intensity of generating ROS/RNS with the activity of oxidant scavengers, which may be overwhelmed due to exercise. Traditionally, ROS/RNS were associated with skeletal muscle fatigue development and damage; however, the role of ROS/RNS in muscle performance is not as straightforward as initially thought. Contrary to massive ROS/RNS synthesis, low-to-moderate concentrations were generally thought to be engaged in adaptations to physical effort [15], which was based on the concept of hormesis. The latest data based on the implementation of blood flow restriction (BFR) training protocols, however, challenged this hypothesis. BFR involves a controlled form of vascular occlusion proximal to muscle, typically using a pneumatic cuff, combined with aerobic or resistance training. During the successive periods of cuff deflation, where blood flow was sustained at a level higher than at rest and convective O_2_ transport in the muscle was maximal, ROS/RNS production was amplified [16,17]. The direct measurement of free radical generation and redox markers confirmed systemic increases in ROS following BFR exercise sessions [18,19]. Such a profound rise in ROS/RNS, associated with local reactive hyperemia, might contribute to increases in muscle mass, strength, and performance [20].

### 2.1. The Sources of Reactive Oxygen and Nitrogen Species (ROS/RNS) in Skeletal Muscle

Skeletal muscle is a heterogeneous tissue composed of three major fiber types that possess certain unique metabolic and contractile features. Specifically, slow-twitch oxidative fibers (type I) have a higher oxidative capacity and a higher fatigue threshold that enables them to support sustained aerobic activity. In contrast, fast-twitch glycolytic fibers (type IIx) exhibit a lower oxidative capacity and enzymatic profile that favor anaerobic metabolism, slower calcium kinetics, faster shortening velocities, and the ability to generate more power than slow-twitch fibers. Fast-twitch oxidative–glycolytic fibers (type IIa) exert higher twitch speeds than type I fibers but are less fatigue-resistant. Such a broad range of capabilities has emerged through the selection of a characteristic molecular profile designated to achieve a particular contractile phenotype for each fiber type. Specifically, myosin heavy chain (MHC) isoforms dictate the ATPase activity of muscle and underly the above classification. The MHC I isoform is characterized by a high ATP hydrolysis power, such that a lower maximum shortening velocity and higher economy of ATP usage are developed in MHC I muscle compared with fast MHC IIa and IIx fibers [21]. Furthermore, MHC-I-rich fibers exhibit a lower maximum Ca^2+^-activated force but a higher Ca^2+^ sensitivity compared with MHC-II-rich fibers [22]. At rest, a certain amount of ROS/RNS is continuously produced in muscle as an important component of normal cell signaling in all fiber types, although the magnitude of this process is the highest in glycolytic fibers [23]. Free radicals consist of atoms or molecules with an unpaired electron in their outer shell, which makes them unstable and highly reactive. This group includes superoxides (O_2_^•−^), oxygen radicals (O_2_^••^), hydroxyl radicals (^•^OH), alkoxy radicals (RO^•^), peroxyl radicals (ROO^•^), nitric oxide (^•^NO), and nitrogen dioxide (^•^NO_2_). Non-radical derivatives include hydrogen peroxide (H_2_O_2_), hypochlorous acid (HOCl), ozone (O_3_), singlet oxygen (^1^O_2_), nitrous acid (HNO_2_), nitrosyl cations (NO^+^), nitroxyl anions (NO^−^), and peroxynitrite (ONOO^−^), and they can easily lead to free radical reactions in living organisms [23].

Davies et al. [24] were the first to report that free radicals are elevated in contracting rat muscles in 1982. In 1978, Dillard et al. observed an increased content of expired pentane, an index of lipid peroxidation, after 60 min of cycle ergometer exercise at 50% VO_2_ max intensity, in humans [25]. The first direct evidence for intramuscular free radical accumulation following exercise in humans was provided by Bailey et al. in 2007 with the use of electron paramagnetic spectroscopy [26]. The rate of ROS/RNS synthesis differs with respect to both the intensity and duration of exercise, which together constitute exercise volume. In general, short-term, low-intensity aerobic exercise (<40% VO_2max_) generates relatively less ROS than moderate-intensity exercise (65–75% VO_2max_). However, it needs to be highlighted that this association was concluded based on the net activity of antioxidant systems and the level of oxidative damage to macromolecules (e.g., increased DNA damage, protein oxidation and lipid peroxidation) in both the blood and active skeletal muscles, not the direct measurement of ROS/RNS content. The assessment of radical expression faces difficulties due to the high reactivity and short half-life of ROS/RNS [27,28]. Furthermore, a higher frequency of exercise (five times per week) was found to more efficiently reduce oxidative stress based on malondialdehyde content and improved mitochondrial oxidation capacity compared with exercise performed three times per week [29]. Other aspects determining the rate of ROS/RNS generation include fluctuations in blood flow and local oxygenation. It was shown that isometric muscle contractions at 60% of maximal voluntary contraction (MVC) occlude blood flow, presumably at the level of lower-order arterioles or the capillary bed. The extent of flow restriction is lower at both 30% and 100% of MVC because of the insufficient tension created and shorter duration of time when contraction can be sustained, respectively. The successive reperfusion period (muscle relaxation) was found to enable oxygen delivery, although it was not linearly dependent on the degree of blood perfusion during sustained isometric contractions and accounted for ~42%, ~22%, and ~22% at 30%, 60%, and 100% of MVC, respectively [30]. Regained oxygenation could precede the production of ROS/RNS and provide time for muscle regeneration and adaptation to exercise. To support this notion, the employment of BFR training protocols in human studies coincided with several improvements, including upregulated glucose extraction, Na^+^/K^+^-pump expression, and K^+^ homeostasis, that delay fatigue [17,31]. Moreover, increased muscle temperatures lead to higher levels of ROS during contractions [32]. Whenever ROS production is managed with a biological system’s ability to readily detoxify ROS, exercise appears to not promote oxidative stress. However, this imbalance occurs as a result of the excess level of ROS/RNS or overridden antioxidant system capacity. It is well-established that acute bouts of endurance exercise in untrained humans and animals result in increases in biomarkers of oxidative stress (e.g., increased protein oxidation and lipid peroxidation) in both blood and active skeletal muscles [33].

The main sources of muscular ROS during exercise remain uncertain due to the difficulties in measuring and quantifying real-time contraction-stimulated ROS production. These difficulties are caused by radicals’ relatively short half-life, instantaneous removal by defense mechanisms, and continuous generation in tissues other than skeletal muscle. Under normal physiological conditions, only 0.1–0.2% of the O_2_ consumed by mitochondria is converted into O_2_^•−^, and that value depends on both metabolic status and the oxidative substrate used [34]. Mitochondria can generate ROS from at least 11 different sites, and conventionally, complexes I (site I_Q_) and III (site III_Qo_) within the respiratory electron transport chain, as well as monoamine oxidases activity, are thought to be the dominant contributors of mitochondrial O_2_^•−^ and H_2_O_2_ during exercise [35]. Results regarding both permeabilized fibers and isolated mitochondria showed that fast-glycolytic muscles emit higher H_2_O_2_ levels than slow-oxidative fibers, most likely as a consequence of a considerably lower mitochondrial ROS/RNS scavenging capacity [36]. Most importantly, under ex vivo conditions resembling mild and aerobic exercise in skeletal muscles, the overall rate of mitochondrial O_2_^•−^ and H_2_O_2_ production was found to account for only 15% of that at rest. This was shown to be related to a transition to so-called state 3 respiration (ADP-stimulated oxidative phosphorylation) associated with a lower protonmotive force and the oxidation of ubiquinone pool, while site I_F_ (with low-capacity to O_2_^•−^ production) was shown to be the major ROS contributor [37]. Moreover, the 66 kD isoform of spontaneous human combustion (shc) adaptor proteins (p66Shc) translocates to the mitochondrial matrix upon exercise, wherein it oxidizes cytochrome c to form H_2_O_2_. p66shc-induced mitochondrial ROS synthesis was shown to be protective in conditions of high levels of cell stress, such as during exercise. This was evident in p66shc^−/−^ mice, which were slightly less fatigued during downhill running than control animals. The knockdown of p66shc, however, did not affect skeletal muscle structure and function at rest [38].

NADPH oxidase (NOX) enzymes, rather than mitochondria, appear to be the predominant contributors of ROS in skeletal muscle. Of the NOX family, NOX1, NOX2 (producing O_2_^•−^), as well as NOX4 and DUOX1/2 (producing H_2_O_2_), have been reported to increase during both high-intensity exercise [39] and moderate-intensity exercise [40]. In the presence of ADP and Fe^3+^, the enzymes transfer electrons from NADPH to molecular oxygen to produce O_2_^•−^ and then H_2_O_2_ [41], and the inhibition of NOX enzymes blocks both basal and stretch/contraction-stimulated skeletal muscle ROS production [42]. The results of a study examining the loss of NOX2 in both acute and repeated exercise implied potential cross-talk between different sites of ROS production in skeletal muscle responses to exercise [43]. It is also likely that NOX2 (present in membrane-enriched protein fractions) and NOX4 (mitochondria) mediate specific signaling pathways via ROS production in different subcellular microdomains. Matrix O_2_^•−^ is possibly more important for stress resistance than intermembrane space ROS [44]. On the other hand, NOX4 is activated following decreases in mitochondrial ATP levels and is involved in the adjustment of glucose and fatty acid oxidation to exercise [45].

Concurrently, human skeletal muscles contain approximately 16 different phospholipase A2 (PLA2) isoforms, both calcium-dependent and calcium-independent, capable of stimulating ROS generation in muscles under rest [46] and exercise conditions [47]. Augmented PLA2 activity (in mitochondria and the cytosol) and arachidonic acid release (i.e., a substrate for several ROS-generating enzyme systems including lipoxygenases) during exercise can stimulate NOX [48,49].

XO has been recognized as contributing to O_2_^•−^ generation in the extracellular space following muscle contraction [50] due to its high expression in endothelial cells and activation in response to shear stresses applied to skeletal muscle cells during exercise [34]. XO is most strongly stimulated under conditions of exhaustive exercise when blood flow does not meet requirements. An increase in XO activity is associated with an accelerated conversion of ATP into AMP and eventually into hypoxanthine. XO then catalyzes the conversion of hypoxanthine into xanthine and xanthine into uric acid [34,51].

Skeletal muscles constitutively express neuronal and endothelial nitric oxide synthases (nNOS and eNOS, respectively), which generate ^•^NO from L-arginine, NADPH and O_2_. Approximately 75% of nNOS is associated with submembrane scaffolds that are part of the dystrophin glycoprotein complex, and the remainder is detected in the mitochondria and the sarcoplasmic reticulum [52]. Both neuronal and endothelial isoforms become activated by increases in free cytosolic Ca^2+^ concentration during contractile activities [53]. The produced ^•^NO is an uncharged and freely diffusible molecule, and it may exert effects over distances exceeding 100 μm [54]. ^•^NO undergoes complex interactions with ROS, including direct quenching by O_2_^•−^, to generate the toxic oxidant ONOO^−^ that damages proteins, lipids, and DNA [55]. Even at high physiological concentrations of SOD, the rate of ONOO^−^ synthesis is 6 times faster than the rate at which SOD creates H_2_O_2_ from O_2_^•−^ [56].

### 2.2. Aerobic Exercise

Despite a 1–3-fold increase in O_2_^•−^ during strenuous exercise, mitochondria only account for a small portion of the ROS generated as a consequence of lowered mitochondrial NADH/NAD^+^ ratios and consequently reduced complex-I-dependent ROS production [41]. Studies based on both human and animal models have shown that the major pool of ROS in strenuous exercises is produced by the action of NOX2 and NOX4 [57]. Importantly, specific mitochondrial phenotypes exist in slow- and fast-twitch fibers in line with distinct patterns of pro-oxidative enzymes [39]. In this case, some specificity in NOX2 and NOX4 responses to exercise was shown between different types of muscle fibers. Particularly, initial NOX activity was significantly higher in the slow-twitch oxidative soleus muscle compared with the fast-twitch oxidative–glycolytic red gastrocnemius and the fast-twitch glycolytic red gastrocnemius [39]. Other biological systems more strongly contributing to exercise-induced ROS production include XO activated by the breakdown of ATP to support repetitive contractions [58].

### 2.3. Anaerobic Exercise

In 2000, ROS/RNS production during and after anaerobic exercise was observed based on a higher lipid hydroperoxide content in the blood following isometric exercise [59], and direct increases in blood free radical levels were later observed in male individuals performing the Wingate test (a 30-s full-strength pedaling exercise) [60]. Further insight into the underlying mechanisms revealed that the main site of ROS/RNS production in the fast-twitch fibers was a cytosolic compartment. These results were also supported by the fact that during short-term anaerobic exercises, only 0.15% of O_2_^•−^ was found to be produced in the mitochondria [61]. Some studies have shown that high-intensity exercise is also coupled with the activation of NOX2 and NOX4 located in the sarcolemma, transverse tubules, and sarcoplasmic reticulum of muscle cells [43,62]. The accelerated ATP degradation and accumulation of downstream metabolites lead to the activation of XO [61], which is directly correlated with lactic acid levels [63]. However, there were no reported differences in the levels of ROS generation between eccentric contractions that damage the muscle and isometric contractions that do not cause injury [64]. Additionally, increased real-time ^•^NO production was visualized in skeletal muscle isolated from mice using a fluorescent ^•^NO probe, 4-amino-5-methylamino-2′,7′-difluorofluorescein diacetate (DAF-FM DA). Five minutes of electrically stimulated contractions led to a marked increase in ^•^NO production in fast-twitch fibers, which rapidly normalized by 15 min post-stimulation [65]. The abrupt nature of RNS changes in skeletal muscle highlights the importance of real-time monitoring systems for the direct detection of ROS/RNS production throughout exercise protocols.

### 2.4. Endogenous Mechanisms of Reactive Oxygen and Nitrogen Species Detoxification

**Figure 1:**
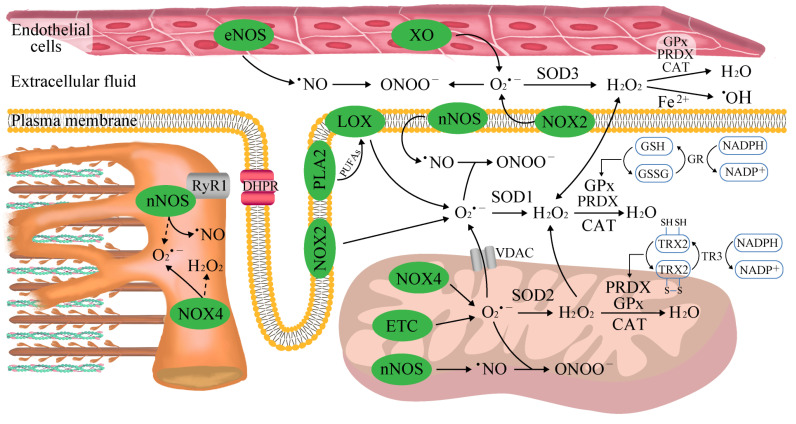
Overview of the major oxidant and antioxidant systems in skeletal muscle. Mitochondrial respiration shifts from state 4 to active state 3 when muscle contractions start, which is characterized by a lower superoxide (O_2_^•−^) production than at rest. NADPH oxidase (NOX) enzymes become activated in response to sarcolemma depolarization, ATP release, and fiber stretching associated with exercise. NOX2 resides in the plasma membrane and transverse tubules, and it is the main source for cytosolic O_2_^•−^ generation in contracting muscle. NOX4 was reported to localize in the sarcoplasmic reticulum and mitochondrial intermembrane space. Xanthine oxidase (XO) is abundant in endothelial cells and activates via increases in shear stresses applied to skeletal muscle and ATP hydrolysis. The release of polyunsaturated fatty acids (PUFAs) from plasma membrane is stimulated by phospholipase A_2_ (PLA_2_), and the released free PUFAs are subsequently oxidized by lipoxygenase (LOX). The formed O_2_^•−^ is detoxified with the assistance of superoxide dismutase (SOD), catalase (CAT), glutathione peroxidase (GPx), peroxiredoxin (PRDX) or thioredoxin (TRX). The PRDX-mediated reduction in H_2_O_2_ uses TRX as the electron donor. In the course of the reaction, one sulfhydryl group of the cysteine residues in PRDX is oxidized and a disulfide bridge is formed. Then, the disulfide bridge is reduced by TRX at the expense of NADPH oxidation. Abbreviations: DHPR, dihydropyridine receptor; ETC, mitochondrial electron transport chain; GR, glutathione reductase; RyR1, ryanodine receptor 1; TR3, thioredoxin reductase 3; VDAC, voltage-dependent anion channel.

Skeletal muscle is a highly plastic tissue, and under most exercise conditions, oxidative balance is preserved within physiological range via the upregulation of ROS scavenging, hence minimizing the potential for oxidative damage. The intensity and duration of physical exercises are critical in this respect. When exercise is being performed on a regular basis, even 5–10 consecutive days of moderate-intensity exercise markedly increase both the oxidative and antioxidant capacity of skeletal muscle fibers, which upregulates fatigue resistance (Figure 1) [66,67]. Similarly, long-term (12 weeks) endurance exercise training augments antioxidant enzyme activities in the muscle and reduces contraction-induced oxidative stress [68]. Increases in the antioxidant enzyme activities in plasma were detected after both maximal and submaximal exercise periods in untrained men [69].

The multifaced antioxidant aperture consists of the three main strategies. First, numerous low-molecular-weight molecules able to scavenge ROS co-exist in the extracellular space and within cells. This group includes glutathione (GSH), uric acid, lipoic acid uric acid, bilirubin, vitamin E, and vitamin C. Second, some enzymatic antioxidants act by converting ROS into less reactive molecules, including copper–zinc superoxide dismutase (SOD1), manganese superoxide dismutase (SOD2), extracellular superoxide dismutase (SOD3), catalase, and glutathione peroxidase (GPX). A final antioxidant mechanism is based on the binding of pro-oxidant transition metals (e.g., iron and copper) via metal-binding proteins. The chelating molecules preclude these transition metals from participating in ROS formation [27].

A distinct phenotypical predominance of exercise-induced antioxidants holds true for SOD, which is most noticeably upregulated in active skeletal muscles composed of highly oxidative fibers (e.g., type I and type IIa) [39,70]. The importance of SOD1 to preserve muscle function has been confirmed by studies employing SOD-deprived mice (*Sod1*^−/−^). *Sod1*^−/−^ animals were found to exhibit a characteristic phenotype with peripheral nerve integrity and denervated motor end plates, which resulted in fiber loss and muscle atrophy [71] and was linked to elevated oxidative damage in DNA, proteins, and lipids, as well as an increase in proteolytic activity [71]. Compared with whole-body *Sod1*^−/−^ strain, muscle-restricted SOD1 deficiency was found to be insufficient to reproduce the accelerated neuromuscular degenerative phenotype, partially through the compensative upregulation of other antioxidative pathways, and these animals maintained levels of muscle mass similar to respective control mice. Nevertheless, both systemic and skeletal muscle-specific SOD1 knockout affected muscle structure, with the relocation of nuclei towards the cell center, hence reflecting continuing cycles of degeneration and regeneration [72]. Similarly, muscle *Sod2*^−/−^ mice showed centralized nuclei in their muscle fibers and the selective loss of respiratory enzyme activities, including complex I and complex II (succinate dehydrogenase), as well as reduced ATP levels in their skeletal muscles. A single dose of the EUK-8 antioxidant significantly improved exercise activity in mice [73], which demonstrated the critical contribution of the O_2_^•−^ generated in mitochondria in the progression of deficits in muscle structure and force generation and the development of exercise intolerance.

The impact of exercise on catalase expression or activity remains controversial, as studies have reported mixed results with respect to aerobic (unchanged) and anaerobic (reduced) training [74,75]. Nonetheless, the increased expression of human catalase specifically targeted to mitochondria in the skeletal muscle of *Sod1*^−/−^ mice rescued from disruptions was associated with mitochondrial dysfunction and neuromuscular junction-related phenotypes, consequently preserving muscle mass and strength [76]. On the other end, the individual transgenic overexpression of catalase, SOD1 or SOD2 failed to delay diaphragmatic fatigue during prolonged submaximal contractions or enhance recovery following an exercise protocol [77]. Altogether, these results are consistent with the hypothesis that contraction-induced ROS/RNS at a certain level are indispensable in the optimization of muscle performance and that strong radical eradication affects both the morphology and function of muscles.

Endogenous thiol antioxidants glutathione (GSH), thioredoxin (TRX) and periredoxins (PRDX) are modulated by oxygen consumption and ROS generation during physical exercise, coupling the intracellular changes in the redox state to the control of cellular processes [78]. GSH is the predominant nonprotein thiol in the cell, with the ratio of reduced to oxidized glutathione (GSH/GSSG) being a primary indicator of the redox state and oxidative stress in multiple modes of exercise [79]. Even though regular exercise might not affect the GSH/GSSG ratio, the post-exercise GSSG level increases as a consequence of the simultaneous exercise-mediated stimulation of GSH synthesis to better cope with anticipated ROS production [80]. More prominent changes are observed when exercise is performed at higher intensities and by untrained individuals. Moreover, both aerobic (cycling) and anerobic (static handgrip and thumb adduction) overtraining were found to predispose participants to decreases in the blood GSH/GSSG ratio, which were highly correlated with drops in performance [81,82]. For example, trained men who exercised to exhaustion on a treadmill had increased blood levels of oxidized glutathione immediately after exercise, but they returned to rest after 1 h [83]. GPx activity, which is critical for both cytoplasmic and mitochondrial ROS detoxification, varies with the predominant fiber type and is most extensively expressed in highly oxidative skeletal muscles [39,84]. In line with these results, the magnitude of exercise-mediated increases in GPx function was found to be proportional to exercise duration in the red gastrocnemius but remained relatively constant in the white gastrocnemius [85]. In this regard, GSH status may affect force generation via direct effects on contractile protein functions. A chemical process, namely, non-enzymatic S-glutathionylation, occurs during thiol-disulfide exchange through the participation of protein thiols (Pr-SH) and GSSG. The S-glutathionylation of a specific cysteine residue (Cys134) present in the highly mobile COOH-terminal domain of troponin I in fast-twitch muscle fibers was observed during strenuous exercise in rats and humans. Cysteine, acting as an antioxidant, can improve Ca^2+^ sensitivity and thereby counteract fatigue by promoting the interaction between troponin I and troponin C at lower cytoplasmic Ca^2+^ concentrations [86,87,88]. The resultant reversible increase in the Ca^2+^ sensitivity of the contractile apparatus was shown to influence muscle performance by offsetting the unavoidable biochemical and ionic changes occurring in the muscle during exercise [86,87]. Diminished force responses and muscle fatigue occur in the face of unrestricted decreases in contractile Ca^2+^ sensitivity. Moreover, a six-week BFR training program was shown to accentuate beneficial ROS-mediated improvements in performance. BFR training resulted in an increase in peak aerobic power output in accordance with the improved redox status determined by an elevated GSH/GSSG ratio. These results coincided with a higher net K^+^ release in response to concomitant antioxidant provision (NAC) in individuals. The study authors proposed that an elevated GSH availability and, hence, enhanced potential for Na^+^/K^+^-pump S-glutathionylation would translate into the accelerated K^+^ re-uptake of muscle and improved cell excitability [89]. Another important observation was the selective activation of cytosolic antioxidants rather than mitochondrial defense systems, which indicates that ROS derived from cytosolic compartments play a more prominent role in the development of fatigue resistance [31]. These data are supported by a study in mice, wherein an increase in cytosolic ROS preceded and overgrew the rise in mitochondrial ROS during contractions [90].

The TRX antioxidant system is composed of cytosolic (TRX1) and mitochondrial (TRX2) isoforms, which possess oxidoreductase activity that maintains intracellular proteins in a reduced state. TRX forms a disulfide bond with the target protein and then transfers two of its electrons to it. To enable continuous antioxidant activity, oxidized TRX is restored to its reduced form by electron transfer from NADPH via TRX reductases (TRs) [91]. Although the loss of cellular TRX1 is known to result in elevated GSH levels, both thiol systems appear to have distinct signaling and control circuits [92]. For instance, the endogenous ROS generated during contractions modulate muscular TRX status. At the same time, GSH and other thiols are oxidized, which indicates that GSH-dependent mechanisms in skeletal muscles are more susceptible to oxidation [93]. On the other hand, TRX does not seem to modulate redox-regulated adaptations to muscle contraction. Recent data revealed that while the TRX system remained unchanged in human skeletal muscle after prolonged aerobic exercise [94], it was increased after repeated sprint bouts [95]. Moreover, plasma TRX levels were found to rise in response to the oxidative stress induced by exercise, which was attributed to TRX expression in peripheral blood mononuclear cells. It is also important that the TRX1 protein can be secreted from skeletal muscle, which also mediates increases in its circulatory activity [96].

PRDX belong to a group of ubiquitous oxidoreductase proteins that contain catalytic cysteine residues and are oxidized to a sulfenic acid (SOH) following the attack of a hydroperoxide substrate. PRDX is a major regulator of mitochondrial H_2_O_2_, likely scavenging up to 90% of the H_2_O_2_ generated within this compartment. The formation of overoxidized PRDX (isoforms I–IV) was noticed in humans in response to a high-intensity trial (80% VO_2max_) compared with 60% VO_2max_ and exceeded the reductive potential of TRX [97]. These sequential patterns of antioxidant activation can partially be associated with the progressing decrease in cellular pH that suppresses the sensitivity of TRX cysteines to oxidation. This so-called “floodgate model” allows the initially formed H_2_O_2_ molecules to modify cell function and growth via the oxidation of other thiol proteins, including protein tyrosine phosphatase 1B (PTP1B), phosphatase and tensin homolog (PTEN) and serine/threonine kinase ATM [98]. So far, mechanistic approaches in knockout models have revealed a principal role of PRDX3 in mitochondrial fusion and ATP production. The ablation of PRDX3 in mice did not affect the maximal contractile force production but did substantially accelerate the rate of muscle fatigue in both the extensor digitorum longus and soleus muscles. Since the effect was more pronounced in slow-twitch fibers, the results confirmed these fibers’ high reliance on appropriate mitochondrial activity in exercise [99]. Affirming these results, PRDX3 overexpression prevented the loss of mitochondrial electron transport chain activity and a decrease in the maximum isometric specific force in SOD1-deprived mice [100]. Furthermore, the loss of PRDX2 in a dystrophin-deficient *mdx* mouse model led to eccentric contraction-induced force loss in the extensor digitorum longus, likely because PRDX2 serves as an off-switch to control stretch-activated NOX2 signaling in healthy skeletal muscle [101]. These results indicate that PRDX could govern the physiological effects of H_2_O_2_ in active muscles during exercise and extend the time to fatigue in contracting muscle.

### 2.5. Reactive Oxygen/Nitrogen Species and Adaptations to Exercise in Skeletal Muscle

**Figure 2:**
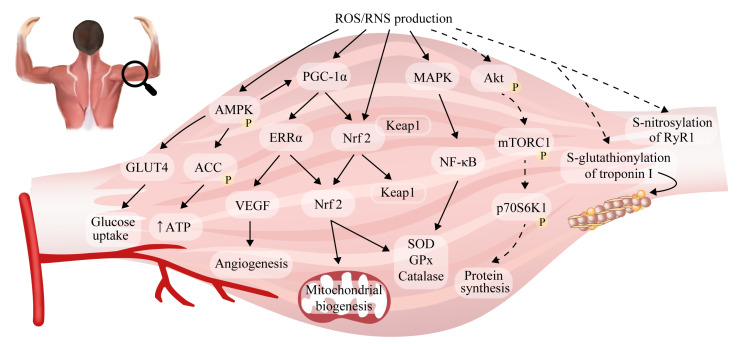
Redox-regulated signaling pathways in muscle adaptations to exercise. During endurance exercise (solid arrows), ROS/RNS participate in AMPK activation to promote glucose uptake, ATP generation, vascularization, mitochondrial biogenesis, and antioxidant defense (via the PGC-1α pathway). Dashed arrows indicate mechanisms that dominate in response to resistance exercise in fast-twitch fibers. ROS/RNS trigger post-translational modifications in ryanodine receptor (RyR1) and troponin I to increase ongoing force production. The long-term effect is the stimulation of mTORC1-p70S6K1 signaling to orchestrate overload-induced muscle hypertrophy. Abbreviations: ACC, acetyl-CoA carboxylase; AMPK, AMP-activated protein kinase; ERRα, estrogen-related receptor α; Keap1, Kelch-like ECH-associated protein 1; MAPK, mitogen-activated protein kinase; mTORC1, mammalian target of rapamycin 1; NF-κB, nuclear factor-kappa B; Nrf2, nuclear factor erythroid 2-related factor 2; p70S6K1, p70 ribosomal protein subunit 6 kinase 1; RyR1, ryanodine receptor 1; VEGF, vascular endothelial growth factor.

Subjects involved in interval training, which is characterized by short periods of high-intensity exercise interspersed with periods of recovery, develop adaptive mechanisms related to not only the renewal of glycogen stores in skeletal muscle but also higher levels of mitochondrial content and the effectiveness of oxidative damage repair systems. Additionally, the subjects produce lower levels of ROS at a given intensity of exercise compared with less-trained individuals [92]. Several important pathways have been proposed in mediating these physiological adaptations to training (Figure 2), although the extent of muscle reprogramming depends on workload and exercise volume [102]. Although some human studies have shown that exercise intensity is the most important variable determining the stimulation of mitochondrial function [103], other research has suggested that exercise training volume is more significant [104].

Fundamental responses to training are increases in mitochondrial respiratory capacity and content, which enhance endurance performance [105]. It is suggested that the ROS generated during regular exercise are prerequisites for the adaptive activation of primary mitochondrial signaling pathways in skeletal muscle [106]. The post-exercise induction of peroxisome proliferator-activated receptor γ co-activator 1α (PGC-1α) was observed in human muscle under both endurance and resistance protocols [107]. The upstream signals that regulate PGC-1α expression such as mitogen-activated protein kinase (MAPK) and nuclear factor (NF)-κB are redox-sensitive [61] and influence muscle response to exercise. Additionally, an increase in PGC-1α in human skeletal muscle was shown to be associated with AMP-dependent protein kinase (AMPK) signaling, which is triggered by increased intracellular ROS concentration and its dependent energy parameters. In turn, AMPK regulates redox balance to a certain degree [108]. Several other oxidative stress biomarkers have been linked to muscle mitochondria function, including heat-shock proteins 27 and 70 (HSP27 and HSP70) and uncoupling protein 3 (UCP3) [109,110]. HSPs are a part of the cellular defense against different stressors and participate in the recovery of muscle function after exercise. They are most significantly upregulated after eccentric high-force contractions and may therefore mediate greater protein remodeling and muscle hypertrophy [109,111]. In turn, a rise in UCP3, subsequent to increased ROS generation, may partially dissipate the proton gradient across the inner mitochondrial membrane, which protects from further ROS generation via the electron transport chain and exercise-induced oxidative stress [110]. The resultant increase in PGC-1α greatly upregulates mitochondrial biogenesis by coordinating the expression of nucleus- and mitochondria-encoded genes [112], as well as by directly antagonizing protein catabolism by suppressing FoxO3, which is responsible for proteasomal and lysosomal protein degradation [107]. Moreover, PGC-1α facilitates the activation of nuclear factor erythroid 2-related factor 2 (Nrf2), which plays an important role in promoting the exercise-induced expression of >200 key components involved in the endogenous antioxidant system [61]. In fact, Nrf2 controls the expression of key components of the GSH and TRX systems as well as enzymes involved in NADPH generation in order to prevent muscle damage [113,114]. PGC-1α has also been implicated in fiber type determination by increasing the proportion of type I fibers. Slow-twitch fibers exhibit higher PGC-1α contents than glycolytic muscles [115]. Accordingly, in PGC-1α-overexpressing transgenic mice, the hypertrophic muscle phenotype was found to correspond with an elevated generated maximum force and a higher fatigue resistance than control animals [107]. Therefore, it can be stated that gene products that restore intracellular oxidant–antioxidant homeostasis and provide protection from possible oxidative stress are often produced when redox-sensitive pathways are activated.

Resistance training is commonly introduced to enhance muscle strength via hypertrophy. Muscle fibers with high oxidative metabolism, especially, are characterized by a considerable capacity for protein synthesis, which is positively correlated with the type I myosin heavy chain. Additionally, high-intensity protein synthesis is associated with a greater rate of amino acid uptake, higher myonuclei number per volume of cytoplasm, and a greater percentage of myonuclei that belong to satellite cells [116]. One of the major factors involved in such mechanisms is the contraction-induced ROS production responsible for the activation of protein kinase B, which promotes protein synthesis in cells via the downstream activation of mammalian target of rapamycin (mTOR) [117]. mTOR sustains mechanical load-induced hypertrophy by stimulating the protein synthesis of contractile elements. In this regard, mTOR deficiency in muscle induces defects in mitochondrial metabolism and a decrease in mitochondrial gene expression. These concepts are supported by previous reports that mTORC1 is a positive regulator of PGC-1α, and hence mitochondrial biogenesis [117]. Additional data revealed that the overload of plantaris muscle with training was linked to the production of ONOO^−^ and triggered a signaling cascade, resulting in the direct activation of mTOR [27].

Another important biologic response triggered by exercise is the enhancement of insulin sensitivity. Because of the increased skeletal muscle insulin sensitivity during exercise, muscles have a higher metabolic flexibility and the ability to restore glycogen for successive bursts of vigorous exercise. Several molecular regulators of insulin sensitivity have been shown to be upregulated in response to exercise-induced ROS formation in humans. One possible reason for this observation is that the ROS-induced oxidation of cysteine residues in Kelch-like ECH-associated protein 1 (Keap1) results in Keap1 dissociation from Nrf2 and the subsequent translocation of Nrf2 to the nucleus. Nrf2 binds to the antioxidant response element (ARE) and controls gene transcription [118]. In line with this hypothesis, GPx1 deficiency and increased H_2_O_2_ generation [119], or the deletion of Keap1 [120], contribute to improved glucose tolerance and insulin signaling in skeletal muscle. Further data from isolated mouse muscles showed that small GTPase Rac1 is activated by muscle contraction and mediates increases in glucose uptake [121]. NOX2-derived ROS appear to a major contributor to these response since exercise-stimulated glucose uptake and GLUT4 translocation were found to be largely mitigated in NOX2 loss-of-function mouse models [40]. Additionally, NOX2-deficient mice displayed blunted high-intensity-training-induced improvements in antioxidant potential (i.e., reduced SOD2 and GPx levels), maximal running capacity and body fat content, which was suggested to be caused by the mitigation of the NF-κB pathway [43]. According to knockdown experiments, the H_2_O_2_ generated by NOX4 in skeletal muscle and subsequent Nrf2 stimulation are essential regulators of antioxidant defense, mitochondrial biogenesis and insulin sensitivity [122]. NOX4-derived H_2_O_2_ was also reported to control cytosolic Ca^2+^ concentration during tetanic contraction to upregulate serine/threonine phosphatase calcineurin, which selectively stimulates slow-fiber-specific gene promoters [122].

In addition, exercise-induced ROS are able to stimulate skeletal muscles to secrete cytokines, so-called “myokines” that play important roles in the regulation of cell signaling in maternal muscles and other tissues [12,123]. For instance, interleukin-15 (IL-15) was identified to as an anabolic factor, highly stimulated in human skeletal muscle after a bout of resistance training [124]. IL-15 controls intracellular ROS production and attenuates oxidative stress in skeletal muscle cells [125].

Furthermore, it was established that ^•^NO can modulate skeletal muscle force production by regulating the gene expression involved in mitochondrial biogenesis. The effect was associated with an increase in guanylate cyclase activity and the cGMP-dependent upregulation of PGC-1α, Nrf1 and Tfam expression, with subsequent increases in coupled mitochondrial respiration and ATP production [126]. The inhibition of NOS limited these adaptive changes (reduced mtDNA, basal O_2_ consumption rate, and ATP level) [126] and attenuated increases in muscle force [127]. Additionally, thiol modifications may underlie the adaptive response to exercise, as they have emerged as post-translational modifications that dictate physiological ROS/RNS consequences. For instance, alterations in ryanodine receptor type 1 (RyR1) can play an integral part in muscle adaptations to exercise. Both NOX-induced ROS (via the oxidation of cysteine thiols) and ^•^NO-mediated S-nitrosylation can activate RyR1. These modifications induce calmodulin dissociation from RyR1 and release the receptor from FK506-binding protein (FKBP12)- and calmodulin-induced inhibition; consequently, the oxidation of RyR1 and calmodulin enhances Ca^2+^ release, which may stimulate excitation–contraction coupling [128,129]. In the presence of oxidative and nitrosative stress, RyR1 is prone to S-glutathionylation that reinforces the receptor’s open conformation by suppressing the inhibitory effect of Mg^2+^ binding to RyR’s activation site [130]. It is also important to note that different fiber compositions contribute to muscle susceptibility to low ^•^NO-induced fatigue resistance. To address this relationship, the loss of nNOS substantially accelerates contraction-induced muscle fatigue and depresses post-exercise force recovery, solely in fast-twitch fibers [131].

Skeletal muscle nutrient and oxygen supplies rely on an intricate network of blood vessels. An increase in muscle vascularization is one of the early steps in muscle adaptation following endurance training and requires a tight balance between pro- and anti-angiogenic factors. The dominant pro-angiogenic mediator is vascular endothelial growth factor (VEGF). Higher ROS/RNS production during conditions of elevated blood flow and shear stress in the vasculature of working skeletal muscle can potentiate this effect by recruiting PGC-1α, which controls *VEGF* and other pro-angiogenic genes [132]. It has also been observed that the ROS/RNS-driven upregulation of NOS expression in endothelial cells can potentiate flow-mediated dilation. Hence, the redox regulation of vascular function has emerged as a potentially important mechanism contributing to exercise adaptation and fatigue resistance [133]. These findings were further supported by a study wherein supplementation with an antioxidant, resveratrol, in men limited increases in muscle VEGF proteins and angiogenesis mediated by training [134].

### 2.6. ROS/RNS as Fatigue Mediators

Contrary to an intermittent regular exercise protocol, wherein exercise-induced ROS production is mostly overcompensated for by an upregulated antioxidant defense, exhaustive exercise or chronic exposure to ROS can exceed the antioxidant barrier and lead to oxidative stress and damage to muscle fibers [33,92]. The first discovery that radicals contribute to muscle fatigue in animals was reported in 1990 [135], and since then, several ROS-triggered mechanisms have been shown to limit muscle performance. The inhibitory effects of O_2_^•−^ and H_2_O_2_ [3], ^•^NO [136], and ONOO^−^ [4] on maximum force production have been shown to be most pronounced in type II fibers. This could be attributed to variations in endogenous ROS/RNS scavenging capabilities; in fact, the substantially higher antioxidative potential of slow-twitch fibers was confirmed in several studies. For instance, an approximately 5-fold higher GSH level was noticed in slow-twitch muscle compared with fast-twitch muscle [36,39,137].

One of the potential mechanisms that contributes to fatigue is a decrease in membrane excitability, which is most pronounced deep within the transverse tubular system [138]. According to research on mechanically skinned rat muscle fibers undergoing fatiguing stimulation, the decreased excitability of transverse tubules is caused by the *S*-glutathionylation of Na^+^/K^+^-ATPase in response to the excessive production of ROS, which might be accelerated by contraction-induced ATP decline and preclude the restoration of the Na^+^/K^+^ balance with repeated contractions [139]. Some basal level of the inhibitory S-glutathionylation of the Na^+^/K^+^-pump β-subunit that intensified during intense exercise, which coincided with fatigue, was also noticed in biopsies of human vastus lateralis muscles [140]. Experimental evidence to support these data was provided following antioxidant *N*-acetylcysteine (NAC) administration in humans. More specifically, NAC supplementation in humans markedly alleviated the percentage decrease in maximal Na^+^/K^+^-pump activity caused by submaximal fatiguing exercise, most likely via the preservation of muscle GSH and cysteine levels, which can prevent the oxidation of SH groups on the Na^+^/K^+^-pump [141], suggesting that ROS/RNS may jeopardize the potential transmission of cellular action. However, another study did not reproduce action potential failure due to H_2_O_2_ exposure in an animal model employing isolated intact mouse flexor digitorum brevis fibers [15]. Similarly, slow-twitch soleus mouse muscle did not exhibit impaired excitability during fatiguing stimulation [142].

One sign of impaired excitation–contraction coupling could be the affected ability of the sarcoplasmic reticulum to load and release Ca^2+^. Indeed, in vitro studies performed with sarcoplasmic reticulum vesicles isolated from fast-twitch fibers reported interrupted Ca^2+^ outflow and contraction failure in response to NOX activation [143]. As previously mentioned, the oxidation and S-nitrosylation statuses of RyR1 sulfhydryl groups are decisive factors in determining the functional activity of the channel [144]. Generally, stimulatory RyR1 oxidation occurs at high O_2_ concentrations and prevents the S-nitrosylation of a separate cysteine thiol that activates RyR1 at low O_2_ levels [130]. A calmodulin-dependent protective effect of S-nitrosylation against the oxidation of RyR1 has also been observed [145]. The use of H_2_O_2_ at high concentrations (10–100 mM) as shown to have a biphasic effect on RyR1 activity, firstly activating Ca^2+^ release and then suppressing the channel as a function of time [146]. The extent of RyR1 S-nitrosylation is limited under physiological conditions through denitrosylation-based mechanisms, resulting in the removal of ^•^NO groups from the RyR1 channel. However, pathologic RyR1 hypernitrosylation induces the leak of Ca^2+^ from the sarcoplasmic reticulum, leading to progressive deterioration in muscle performance [147]. The suppressive role of RyR oxidation was also noticed in skinned fibers prepared from the isolated extensor digitorum longus muscle of a rat, wherein the O_2_^•−^ scavenger Tiron mitigated an uncoupling between Ca^2+^ transport and the sarcoplasmic reuptake mediated by Ca^2+^-ATPase at higher temperatures [148]. Despite these findings, exogenous ROS exposure generally has negligible effects on the amount of Ca^2+^ released from the sarcoplasmic reticulum in response to action potential in intact rat preparations [3] and mouse fibers, even at higher temperatures [142]. These data reveal that though Ca^2+^ release is sensitive to ROS when artificially triggered in skinned fibers, it is not responsive to nervous stimulation. During exercise, RyR1 is progressively oxidized, S-nitrosylated, hyperphosphorylated, and depleted of inhibitory counterparts (e.g., FKBP12), eventually triggering the “leakiness” of a channel and decreasing exercise tolerance [149]. The described dose-dependent pattern of RyR inhibition at high but not low ROS levels (inverse U-shaped model) highlights the roles of oxidative stress rates and duration in muscle fatigue regulation [150]. Moreover, ROS/RNS suppress the sarcoplasmic endoplasmic reticulum Ca^2+^-ATPase (SERCA) by modifying the sulfhydryl groups of the SERCA protein or by directly interfering with the ATP-binding site [151]. Overall, ROS-mediated fatigue in the course of exercise targets both RyR1 and SERCA to affect Ca^2+^ homeostasis.

More current evidence suggests that ROS/RNS-mediated fatigue may not be due to a loss of membrane excitability or an inability to release Ca^2+^; rather, it may be caused by downstream Ca^2+^ signaling. Specifically, the role of H_2_O_2_ exposure in the modulation of contractile function and acceleration in force decline during submaximal tetani was found to be accompanied by a decrease in myofilament Ca^2+^ sensitivity in intact single murine fibers. Because dithiothreitol (DTT), a disulfide-reducing agent, reversed the H_2_O_2_-triggered muscle strength decline, the reversible oxidation of regulatory thiols in myofibrils was most likely the main mechanism through which ROS induced fatigue in the isolated murine muscles [152,153]. Generally, ROS/RNS can change the primary structure of proteins and contribute to their secondary and tertiary structure modifications so that their polypeptide chains unfold to form random structures. Multiple lines of evidence confirm these assumptions. For instance, O_2_^•−^ exposure was shown to decrease maximum force, but it had no effect on the amount of Ca^2+^ (pCa_50_) needed to achieve half the maximum force in rabbit diaphragm fibers [154]. Accordingly, the reason for the greater rate of fatigue at 37 °C compared with 22 °C was a large decrease in mouse myofibrillar Ca^2+^ sensitivity. In the presence of Tiron, a decrease in Ca^2+^ sensitivity was markedly attenuated and a slower decline in tetanic cytosolic Ca^2+^ was observed [5]. Therefore, by causing a reduction in myofibrillar Ca^2+^ sensitivity, the combination of elevated temperature and Fe^2+^ had significant force-decreasing properties. This effect was most likely mediated by the OH^•^ generated from H_2_O_2_ in the Fenton reaction [155]. The role of OH^•^ as a main fatigue-mediator is also supported by the substantial exacerbation of Ca^2+^ desensitization in the contractile apparatus in response to H_2_O_2_ and myoglobin co-application, since H_2_O_2_ reacts with Fe^2+^ in myoglobin [156]. The contractile apparatus of fast-twitch fibers can also be affected by ^•^NO donors, independently of H_2_O_2_, as a similar impediment in Ca^2+^ sensitivity was noticed under nitrosoglutathione (GSNO) exposure. In contrast, no perturbations in the force generated by slow-twitch fibers occurred [157]. These observations further explain the higher resistance of slow-twitch muscle fibers to fatigue development and underscore the more prominent role of metabolic factors (e.g., increased inorganic phosphate) in fatigue progression under normal circumstances. The mechanisms for fatigue also appear to differ with respect to antioxidant potential in animal experiments. Prolonged low-frequency force depression (PLFFD) within the flexor digitorum brevis was associated with a decreased sarcoplasmic Ca^2+^ release at a low SOD activity (wild-type mice) or reduced myofibrillar Ca^2+^ sensitivity (SOD2-overexpressing mouse model) [158]. The antioxidant capacity (i.e., higher SOD expression in rats than mouse fibers) and thereby redox status were also proposed as underlying causes for the decreased Ca^2+^ sensitivity observed in rats’ flexor digitorum brevis muscles [158]. Because other authors have also observed a relationship between ROS and reduced Ca^2+^ sensitivity in mouse fibers [5], further mechanistic analyses are required to explain these inconsistencies.

Further data suggest a competitive action of the S-nitrosylation and, as mentioned earlier, S-glutathionylation of cysteine residues in troponin I. In rat fast-twitch fibers, S-nitrosylation treatment was found to be sufficient to block the subsequent S-glutathionylation of this site and mitigated most of its positive effects on Ca^2+^ sensitivity. The reverse was also true, as the S-glutathionylation of Cys134 prevented its S-nitrosylation [88]. The effect of these conditions on force generation during exercise is expected to depend on the extent of S-nitrosylation and S-glutathionylation processes, which are themselves regulated by the quantities of ROS and RNS production and their migration toward troponin complexes. Another problem is the longevity of the observed effects. The study mentioned above only focused on the short-term consequences of post-translational troponin I modifications, so the longitudinal consequences of such a treatment are to be revealed and may differ in future studies. Accordingly, long-term S-glutathionylation reduces Ca^2+^-sensitivity and muscle force [156], and it may depend on the modulation of other residues. The relative role of these processes in specific exercise needs clarification.

Other experiments on rat extensor digitorum longus fibers revealed that a decline in maximal isometric force at higher temperatures might occur in the presence of high O_2_^•−^ concentrations, though independently of Ca^2+^-sensitivity alterations [113,159]. These results imply that ROS may directly affect actomyosin dynamics and cross-bridge attachment complexes rather than regulatory proteins such as troponin and tropomyosin. These impacts may be particularly important because, in the later stages of exhaustion, Ca^2+^ release from the reticulum is hampered by mechanisms unrelated to ROS, resulting in a decreased intracellular concentration of Ca^2+^ at any given stimulation level [160]. In a further analysis, skinned muscle fibers treated with high concentrations of oxidizing substances (DTNB or H_2_O_2_) resulted in functional and structural changes in myosin. More specifically, the use of myosin expressed in *Dictyostelium discoideum* (*Dicty*) showed that the sulfhydryl groups located on the myosin heads were especially sensitive to oxidation. The oxidation of a single methionine residue (Met394) in the actin-binding interface was shown to be directly linked with reduced myosin ATPase activity and diminished maximal Ca^2+^-activated force, which was reversed with a reducing agent, dithiothreitol (DTT) [161,162]. The reversal of the depressive effects of oxidation also occurred after the application of methionine sulfoxide reductase targeting the Met394 residue, confirming the relevance of this particular site in myosin function [163]. The oxidative modification of myosin heavy chains was also noticed in hyperthyroid rats’ soleus muscles, where it led to reduced force production [164]. Despite the above-described susceptibility of myosin to oxidative modifications, the actual mechanisms underlying ROS/RNS effects on cross-bridge kinetics during fatigue remain to be established.

As mentioned above, exercise-induced inflammation plays a crucial role in muscle regeneration. For instance, the NF-κB signaling pathway is activated in a redox-sensitive manner, and then NF-κB selectively binds to DNA response elements and regulates the transcription of antioxidative enzymes in human skeletal muscle [165,166]. However, prolonged or high-intensity exercise is linked with substantial inflammatory responses that could augment mitochondrial dysfunction and ROS production, as well as precede muscle damage [167]. The rise in plasma chemokines after acute bouts of exercise implies an increase in muscle tissue infiltration with monocyte-lineage cells and lymphocytes [33]. It is therefore important to maintain short-term periods of rest between repeated exercise since this blunts the changes in circulating cytokine expression. This beneficial pattern has been noticed in young men and outlines the regular exercise capacity to limit macrophage mobilization [168]. Of note, exhaustive endurance or resistance submaximal exercise can temporarily suppress the immune system (i.e., a reduction in CD4/CD8) [169]. The premature ageing of T cells in athletes raises questions about the potential negative health effects of continuing high-intensity exercise [170].

Additionally, once an imbalance between antioxidant strategies and ROS/RNS arises, a change in macromolecule structure becomes apparent. Lipid peroxidation, protein oxidation, and nucleic acid oxidation particularly occurs as a result of an increase in exercise intensity; these effects can be noticed immediately or even hours after exercise. The lipid peroxidation caused by an excess of endogenous ROS can alter a membrane’s fluidity, permeability, and liquidity, which then causes membrane malfunction. Lipid bilayers are especially rich in polyunsaturated fatty acids (PUFAs), wherein hydrogen abstraction from a carbon and substitution by oxygen result in the generation of lipid peroxyl radicals (LOO^•−^) and hydroperoxides (LOOH) [171]. Finally, the ROS/RNS-dependent fragmentation of RyR1 and increased sarcoplasmic Ca^2+^ leak, which were found to occur in the muscles of recreationally active subjects exposed to high-intensity exercise, depressed force production. These events were blunted in elite endurance athletes and after antioxidant NAC administration [172]. If elevated Ca^2+^ levels persist (24 h) in response to both prolonged medial-intensity exercise and eccentric exercise, a decrease in force production could coincide with the substantial activation of proteases such as calpains. Calpains mainly localize at the I band and Z disk regions of myofibrils and facilitate the turnover of the cytoskeletal proteins responsible for maintaining the structural integrity of muscle, and their overactivation leads to muscle atrophy through the ubiquitin-proteasome pathway and Akt phosphorylation [173]. These processes are often referred to as “low-frequency fatigue”, in contrast with the “high-frequency fatigue” associated with sprint and endurance exercise. In the latter mechanism, Ca^2+^ levels are not elevated long enough to markedly autolyze calpains, so force production can be regained within 30 min [174].

**Figure 3:**
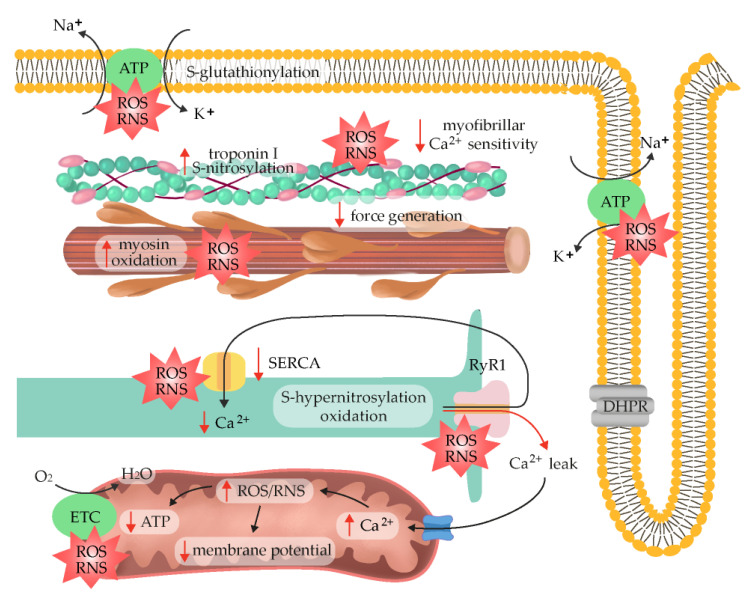
Mechanisms underlying muscle fatigue development in response to high intramuscular ROS/RNS concentration. Increased ROS/RNS levels can influence muscle force production through: (1) enhanced extracellular K^+^ and reduced membrane excitability, (2) the oxidation/nitrosylation of RyR1 and sarco(endo)plasmic reticulum Ca^2+^ ATPase (SERCA) to affect Ca^2+^ release from the sarcoplasmic reticulum and clearance in skeletal muscle, (3) changes in Ca^2+^-sensitivity and cross-bridge kinetics, (4) mitochondrial Ca^2+^ overload and mitochondrial ROS emission, and (5) lowered ATP production.

The described data indicate several target points through which ROS/RNS can impede skeletal muscle force generation (Figure 3). Collectively, the dual consequences of ROS/RNS in muscle performance can be described with a bell-shaped dose–response curve, where a low dose of activity facilitates adaptation and excessive exercise is harmful. Moderate exercise appears to extend the levels of tolerable ROS due to higher antioxidant enzyme activity, thus increasing physiological functioning [150]. However, data from diabetic individuals challenged this assumption, showing that both high (exercise-induced) and low (antioxidant intervention) ROS concentrations can trigger beneficial responses as long as they do not override the redox balance threshold range [175]. Currently, there is no unambiguous protocol or biomarker that can differentiate moderate and excessive exercise and predict either healthful effects or reduced muscle performance.

### 2.7. Exogenous Antioxidants

The mechanistic findings presented above provide a basis for nutritional supplementation strategies or pharmacological manipulations in order to prevent fatigue development. Exogenous antioxidant delivery modifies multiple aspects of skeletal muscle signaling during and immediately following a single bout of exercise, and this can include the modulation of mitochondrial biogenesis, glucose uptake, force production and cell excitability. It has become common practice for athletes and health-conscious, physically active individuals to chronically supplement their normal diets with high doses of antioxidants including vitamins C and E, coenzyme Q10 (CoQ10), α-lipoic acid, and NAC [176]. However, at present, there is not enough evidence to support a role for antioxidants supplementation in preventing the cumulative effects on skeletal muscle caused by radicals during exercise, and this practice may actually hamper certain adaptations to exercise training [57].

Among the most well-known and prevalent antioxidants, vitamins can be easily obtained through natural foods such as vegetables and fruits [177]. The potential prophylactic effects of antioxidant vitamins have been evaluated in an abundance of animal and human studies. He et al. showed that short-term concomitant vitamin C and E administration not only attenuated levels of creatine kinase (a muscle damage biomarker) and muscle soreness but also enhanced muscle protection following a second bout of aerobic exercise [61]. Likewise, Fogarty et al. reported that exhaustive exercise-induced lipid peroxidation and DNA damage can be mitigated by both the short- and long-term consumption of watercress, which is rich in lipid-soluble antioxidants, such as α-tocopherol, β-carotene, and xanthophyll [28]. Despite the beneficial effects mentioned above, a thorough understanding of the application of vitamin and antioxidant supplements such as the optimal dosage, duration, and administration method is necessary to avoid unfavorable effects. Several studies have indicated that antioxidant supplements fail to protect against damaging effects of oxidative stress such as exercise-induced lipid peroxidation and inflammation, both of which hamper muscle recovery. Specifically, prolonged antioxidant ingestion can disrupt endogenous antioxidant levels and impede exercise-induced adaptation, thereby blunting the body’s defense against oxidative stress [178]. Studies on vitamin E have reported the acceleration of oxidative stress in exercising participants following the stimulation of lipid peroxidation and inflammatory process [179,180]. To monitor the effects of ascorbic acid supplementation on post-exercise recovery, muscle strength and redox status were measured 14 days after downhill running. Participants in both groups experienced delayed-onset muscle soreness (DOMS), but those receiving vitamin C also showed delayed muscle recovery [181]. Most importantly, the daily ingestion of vitamin C and vitamin E was found to almost completely blunt mitochondrial adaptive responses by abolishing the changes in PGC-1α gene expression and increases in insulin sensitivity elicited by physical exercise [182,183]. Soon after, Gomez-Cabrera et al. reported that 8 weeks of vitamin C supplementation prevented training-induced mitochondrial biogenesis by suppressing the expression of SOD and GPx [176]. One research showed that consuming large dosages of vitamin C (500 mg kg^−1^) for 14 days inhibited the hypertrophy of overworked muscles in Wistar rats. This event was accompanied by reductions in the ROS-regulated p70S6K and ERK1/2 (regulators of skeletal muscle hypertrophy), although the level of oxidative stress markers was similar between groups. Most likely, the long gap between tissue sampling and the last vitamin C delivery affected the measurements, indicating the transient nature of antioxidant impacts on redox balance [184]. In research by Theodorou et al., males performed eccentric exercise twice a week for four weeks. Concomitantly, they were given vitamins E and C (400 IU and 1000 mg per day, respectively) for 11 weeks, but the high antioxidant doses were unable to improve the antioxidant status (GSH and catalase) of blood and skeletal muscle during and after exercise. The vitamins likely hindered any effect of antioxidant supplementation, so these supplements had no impact on muscular function or post-exercise recovery [185]. On the other hand, an oral antioxidant cocktail of vitamins C, E, and α-lipoic acid (αLA) attenuated circulating free radicals during exercise but impaired exercise-induced brachial artery vasodilation [186]. Following 6 weeks of eccentric leg exercise training, the effects of the administration of αLA and oral vitamins C and E on resting blood pressure and brachial artery vasodilation were assessed via the flow-mediated dilation evoked by both post-cuff occlusion hyperemia and during progressive handgrip exercise. Antioxidants limited free radical concentration, negated training-induced improvements in resting and exercising arterial blood pressure, and significantly decreased flow-mediated vasodilation [187]. Similar findings in isolated rat arterioles confirmed the critical roles of ROS and RNS in flow-induced vasodilation [188], and antioxidants were found to prevent that effect.

αLA can provide effective protection against oxidative stress via a thiol/disulfide exchange mechanism. The compound is unique among antioxidants because it retains powerful antioxidant abilities in its oxidized (αLA) and reduced (dihydrolipoic acid, DHLA) forms. Multiple mechanisms are implicated in αLA’s and DHLA’s antioxidant power, including direct ROS/RNS quenching actions, metal-chelating properties, and the regeneration of endogenous antioxidants such as GSH, vitamin C, and vitamin E [189]. Exercise training and αLA have synergistic effects on improving skeletal muscle glucose transport activity, whole-body glucose tolerance, and lipid profile [190,191]. Athletes supplemented with αLA at a dose of 1200 mg/d for 10 days before exercise showed reduced levels of inflammatory cytokines because of changes in thiol redox status [192]. αLA can have positive effects on maintaining muscle force and reducing muscle damage and inflammation by downregulating the expression of redox-sensitive proinflammatory cytokines, such as TNF-α, Il-6, and inducible NOS. These positive outcomes were associated with training, though not when acute resistance exercise was performed [193]. In other research, no differences in force generation between control and αLA groups were noticed in isolated fibers [194]. Because strategies employing αLA in the prevention of muscle fatigue are in their infancy, there is insufficient evidence to support chronic antioxidant delivery.

Several other compounds have been examined to improve muscle performance, and initial results have indicated beneficial potential. Ubiquinone-10 (CoQ10) is a lipid-soluble compound expressed in high concentrations in meat and fish that exhibits beneficial effects on oxidative stress [195] and inflammatory markers [196]. CoQ10 plays a crucial role in inhibiting the oxidation of the lipids present in membrane structures [197]. No uniform pattern in CoQ10′s impact on endogenous antioxidant levels has been observed; for instance, CAT ↑, GPx 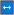 [198] and GPx ↓, SOD 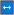 were noticed, while exercise performance measures remained unchanged [199]. However, the use of higher doses of 100 and 300 mg/d suggested that CoQ10 can improve subjective fatigue sensation [8]. CoQ10 also exhibited therapeutic effects against fatigue in statin-treated myopathy and fibromyalgia patients [200]. A recent meta-analysis of the combined results of 13 trials revealed a statistically significant reduction with regard to fatigue symptomology after CoQ10 administration [201]. However, moderate-to-high heterogeneity was observed, especially in relation to the administered dose. A greater exercise capacity was also observed in individuals with mitochondrial disease, while healthy subjects exhibited a greater increase in anaerobic performance in a group without additional CoQ10 supplementation [202]. Therefore, there is no convincing evidence to support the anti-fatiguing properties of CoQ10.

NAC is an acetylated cysteine residue that acts as a ROS/RNS scavenger, and it has been widely tested and approved for use in humans. The ability of NAC infusion to reduce or attenuate muscular fatigue and improve the redox status of cells has been well-recognized. Specifically, NAC supports the cellular resynthesis of GSH and reduces oxidative stress and protein thiol oxidation in the skeletal muscles of humans and mice [203,204]. In NAC-treated rats, an increased tolerance to respiratory muscle loading was observed [205]. Similarly, the same infusion rate of NAC (150 mg kg^−1^) during the low-frequency electrical stimulation of the ankle dorsiflexors was found to enhance force output by 15% in humans [206]. Furthermore, the intravenous infusion of NAC at a dose of 150 mg kg^−1^ prior to an exhaustive exercise at 92% VO_2max_ extended the time to fatigue by 26% in subjects with a superior training status and a higher GSH content [207]. Importantly, the training status of a subject greatly affects muscle responsiveness to this antioxidant, because the VO_2_ peak correlates with the time to fatigue in NAC trials. In line with these results, NAC did not exhibit positive effects on muscle performance in untrained individuals [208]. Intravenous NAC injection also depressed beneficial ROS-mediated increases in glucose extraction in BFR-trained legs [31], an effect that could accelerate fatigue development due to mismatched energy supplies. Since the same effect did not occur in control conditions (without NAC administration), the effects of antioxidants strongly depend on the trained status of skeletal muscles [31]. It was also shown that NAC significantly extended the time to exhaustion at 80% of maximal power in an incremental ramp test but not at higher intensities [209]. Accordingly, in a study by Matuszaczak et al. [203], an infusion of 150 mg kg^−1^ of NAC delayed fatigue during repetitive submaximal efforts but had no effect on muscle force generation during sustained maximal handgrip contractions. Additionally, the chronic administration of NAC may not be beneficial [210].

Contradictory results were also obtained with the use of an XO inhibitor, allopurinol. In the short-term, both NAC and allopurinol alleviate the muscle damage and lipid oxidation induced by acute exhaustive exercise [210]. Allopurinol administration caused an increase in the maximal isometric force of the gastrocnemius muscle in aged mice [211], but XO inhibition did not improve fatigability in young animals [211], instead contributing to the decreased maximum tetanic force produced during nondamaging activity [212]. Two studies have established that allopurinol attenuates [176] or abolishes [50] the increase in skeletal muscle phosphorylation of the p38 mitogen-activated protein kinase (p38 MAPK) during an acute bout of exercise. These findings have implications for the regulation of exercise-induced mitochondrial biogenesis, since exercise-induced increases in the phosphorylation of p38 MAPK are implicated in the activation of skeletal muscle mitochondrial biogenesis [213]. Further supporting the role of XO-produced ROS in mitochondrial biogenesis is that allopurinol was also shown to thwart exercise-induced increases in mitochondrial biogenesis markers such as PGC-1α, as well as mitochondrial transcription factor A (mtTFA) and NRF1 [214], and increases in antioxidant enzymes during acute exercise [50,176].

Pycnogenol (PYC) is a mixture of procyanidins extracted from the bark of the pinus maritime pine, which has antioxidant capabilities. Studies have shown that PYC contains many constituents with free radical scavenging properties, including polyphenols and flavonoids. Specifically, PYC inhibits free-radical-producing enzymes and upregulates the expression of antioxidant enzymes, which translates in ascorbyl radical regeneration and the protection of endogenous vitamin E and GSH from oxidative stress [215,216]. Additionally, a study performed by Mach et al. examined the effect of the supplementation of Lactaway^®^ containing PYC and demonstrated a 17% increase in the time to fatigue during short intensive exercise in humans. It was accompanied by an elevated serum NAD^+^ levels consistent with lower oxidative stress-induced suppression of aerobic respiration due to higher efficiency of electron transfer and ATP production [215].

Sulforaphane, a phytochemical contained in cruciferous vegetables, is an indirect antioxidant that activates the Keap1/Nrf2/ARE signaling pathway under oxidative and/or electrophilic states to exert a cytoprotective effect against oxidative stress [217], and that downregulates NF-κB to suppress inflammation [218]. Accordingly, sulforaphane increased running distance to exhaustion in *Nrf2*^+/+^ mice compared with *Nrf2*^−/−^ mice via the reduction of oxidative stress caused by fatiguing exercise [219]. The continuing sulforaphane intake from 2 weeks before and up to 4 days after eccentric exercise suppressed oxidative marker contents, muscle soreness and damage in humans [220].

Supplementation with GSH has met with little success, as the bioavailability of GSH is extremely low due to the hydrolysis of the tripeptide by intestinal *γ*-glutamyltransferase upon ingestion. Currently, oral formulations of GSH that withstand intestinal breakdown are in demand to counteract this shortcoming. Nevertheless, research involving GSH strongly suggests that it has an important role in preventing oxidative stress-associated lipid peroxidation, which makes maintaining an optimal balance of GSH necessary for it to effectively quench the peroxidation of lipid membranes [83]. In a mouse experiment, 2 weeks of GSH supplementation resulted in elevated concentrations of PGC-1α and mitochondrial DNA in skeletal muscle and prevented exercise-induced reductions in intermuscular pH. These observations suggest that GSH induces aerobic metabolism, which prevents exercise-induced fatigue [221]. Additionally, reduced fatigue-related marker levels were noticed both during and after exercise following GSH supplementation in humans [221].

Resveratrol is a naturally occurring polyphenol that exerts antioxidant effects through Nrf2 activation, and it has been tested in a limited number of human studies with respect to muscle fatigue. Because equivocal results have been obtained, there is no conclusive evidence to recommend its supplementation to athletes. When combined with endurance exercise, resveratrol modestly enhanced indices of cardiac function, mitochondrial density, and muscle fatigue resistance [222,223]. In contrast, 8 weeks of high-intensity exercise training with 250 mg/d of resveratrol administration did not elicit metabolic improvements in healthy aged men. In fact, resveratrol impaired the observed exercise training-induced adaptations in biomarkers of oxidative stress and inflammation in skeletal muscle [224], induced vasoconstriction, and diminished VO_2max_ [225]. It should be noticed that resveratrol also did not improve muscular mitochondrial function markers in a resting state, although higher SOD2, diminished H_2_O_2,_ and lipid peroxidation levels were detected in muscle samples [226], indicating a possible mismatch between the ROS/RNS required for fatigue delay and their observed amount.

## 3. Central Fatigue

Cognitive or perceptual aspects of neuromuscular tiredness are classified as central fatigue and support the brain’s role as an integrative center for signals arising from multiple systems during exercise. The miscalibration of the premotor cortex and the right anterior insula were found to be decisive in subjective effort valuation and willingness to engage in effortful behavior [227]. This may be associated with alterations in the release of neurotransmitters, such as serotonin (5-HT), noradrenaline, γ-aminobutyric acid, and dopamine, that influence the suppression of neural drive to muscles, which translates into decreases in the frequency and synchronization of the motoneurons responsible for the diminished voluntary activation of muscles. At least partially, this can be interpreted as an adaptive mechanism thought to reduce physical exertion or its intensity and to prevent the confinement of exceeded physiological adjustments [228]. Other authors have suggested a role for increasing brain branched-chain amino acid (BCAA) oxidation with exercise progression as a cause for reduced serotonin generation since BCAAs compete with circulating tryptophan to overcome the blood–brain barrier; however, this effect is contradicted by the concomitantly reduced central uptake of tyrosine, a precursor for endurance-stimulating transmitters, such as norepinephrine [229,230]. Additionally, reduced dopamine release and increases in adenosine content are responsible for an increased perception of effort and a reduced motivation to exercise [231]. The contribution of central and peripheral fatigue components differs with the mode and intensity of exercise, with the higher involvement of central nervous system fatigue in aerobics-based, ultra-endurance events compared with shorter exercise events [232,233,234].

The highly oxidative metabolism and high contents of polyunsaturated fatty acids and ROS-active metal ions (such as iron or copper) in the brain make it susceptible to oxidative damage. The velocity and volume of cerebral blood flow are relatively constant and well-protected during exercise, so a reduction in tissue oxygenation does not appear to pose a limiting factor to exercise performance. The development of oxidative stress could be a consequence of the low expression of antioxidant systems in neurons compared with more resilient astrocytes [235]. The available literature suggests that exercise divergently affect antioxidant barriers in specific brain regions, and in structures such as the corpus striatum, stem, and hippocampus, exercise has been shown to upregulate SOD1 and GPx activities [236,237], possibly via mechanisms downstream to pAMPK and PGC-1α stimulation [237]. In agreement with these results, a single bout of exercise was shown to be sufficient to alleviate the oxidative damage of macromolecules triggered by the immobilization of a rat’s hippocampus [238], whereas vitamin C supplementation abated this adaptive mechanism during chronic exercise and caused the oxidative damage of lipids [239]. Among trophic factors, brain-derived neurotrophic factor (BDNF) is induced by myokines released from contracting muscles, such as irisin, and by (at least in neuronal cultures) elevated ROS/RNS levels. In turn, BDNF induces neuronal antioxidant responses through Nrf2 nuclear translocation in an ERK1/2- and PI3K-dependent manner [240], modulates synaptic plasticity, and improves dopaminergic neurotransmission [241], thus possibly delaying the onset of fatigue.

## 4. Conclusions

**Figure 4:**
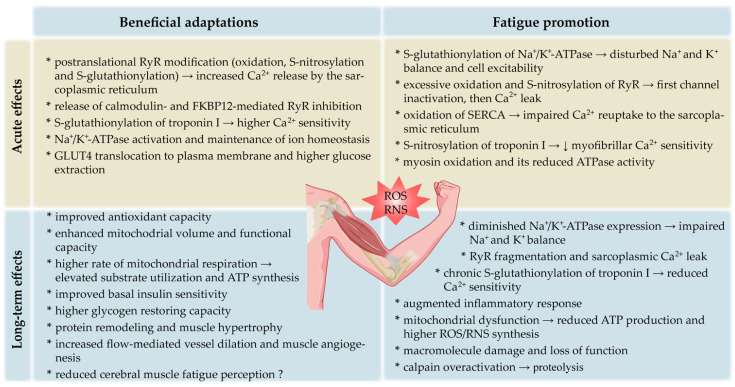
A summary of immediate and long-term ROS/RNS-mediated effects on contractile properties. FKBP12, 12 kDa FK506-binding protein; RyR, ryanodine receptor.

In this review, we summarize the effects of regular and single-bout exercise in terms of oxidative stress and its implications on muscle fatigue (Figure 4). It is clear that ROS/RNS interact at multiple steps of muscle performance at both central and peripheral levels to accommodate applied exercise volumes. Despite a constantly expanding body of evidence linking oxidative stress and muscle force, there are still open questions in the field about the role of ROS/RNS in the intramyocellular modulation of kinase and phosphatase activity, which affect cross-bridge formation and the kinetics of changes in oxidative stress in ongoing exercise, as most studies have generally focused on the isolated muscle and post-exercise changes in redox status. The presented data on BFR training have challenged the hermetic theory assuming that low rates of ROS/RNS production are linked with fatigue delay while reductions in force output result from high increases in ROS/RNS production. The transient surge in ROS/RNS in the reperfusion period has been connected with greater levels of glucose extraction, improved ion homeostasis, and cell excitability that delay the time to fatigue. Importantly, because ROS/RNS are mediators in skeletal muscle adaptations to exercise, the chronic supplementation of antioxidants might prevent the beneficial effects of exercise, including mitochondrial biogenesis and hypertrophy. However, the available literature do not indicate any clear association between exogenous antioxidant provision and changes in endogenous scavengers or fatigue development. Overall, an optimal regime for oxidant scavenger provision, considering exercise volume and subject status (untrained or trained), has yet to be established.

## Data Availability

Not applicable.
